# IL-1α Expression in Pancreatic Ductal Adenocarcinoma Affects the Tumor Cell Migration and Is Regulated by the p38MAPK Signaling Pathway

**DOI:** 10.1371/journal.pone.0070874

**Published:** 2013-08-12

**Authors:** Vegard Tjomsland, Linda Bojmar, Per Sandström, Charlotte Bratthäll, Davorka Messmer, Anna Spångeus, Marie Larsson

**Affiliations:** 1 Molecular Virology, Department of Clinical and Experimental Medicine, Linköping University, Linköping, Sweden; 2 Division of Surgery, Department of Clinical and Experimental Medicine, Linköping University, Linköping, Sweden; 3 Division of Oncology, Kalmar Hospital, Kalmar, Sweden; 4 Moores Cancer Center, University of California San Diego, La Jolla, California, United States of America; 5 Division of Internal Medicine and Department of Endocrinology, Department of Medical and Health Science, Linköping University, Linköping, Sweden; Technische Universität München, Germany

## Abstract

The interplay between the tumor cells and the surrounding stroma creates inflammation, which promotes tumor growth and spread. The inflammation is a hallmark for pancreatic adenocarcinoma (PDAC) and is to high extent driven by IL-1α. IL-1α is expressed and secreted by the tumor cells and exerting its effect on the stroma, i.e. cancer associated fibroblasts (CAF), which in turn produce massive amount of inflammatory and immune regulatory factors. IL-1 induces activation of transcription factors such as nuclear factor-κβ (NF-κβ), but also activator protein 1 (AP-1) via the small G-protein Ras. Dysregulation of Ras pathways are common in cancer as this oncogene is the most frequently mutated in many cancers. In contrast, the signaling events leading up to the expression of IL-1α by tumor cells are not well elucidated. Our aim was to examine the signaling cascade involved in the induction of IL-1α expression in PDAC. We found p38MAPK, activated by the K-Ras signaling pathway, to be involved in the expression of IL-1α by PDAC as blocking this pathway decreased both the gene and protein expression of IL-1α. Blockage of the P38MAPK signaling in PDAC also dampened the ability of the tumor cell to induce inflammation in CAFs. In addition, the IL-1α autocrine signaling regulated the migratory capacity of PDAC cells. Taken together, the blockage of signaling pathways leading to IL-1α expression and/or neutralization of IL-1α in the PDAC microenvironment should be taken into consideration as possible treatment or complement to existing treatment of this cancer.

## Introduction

A highly inflammatory environment is a hallmark for the gastrointestinal malignancy pancreatic adenocarcinoma (PDAC) including a rapid progression and a 5 year survival rate of less than 5% [1], [2]. A massive fibrotic stroma encloses and infiltrates the malignant cells [3] and the cellular composition of PDAC microenvironment supports the recruitment of infiltrating immune cells such as T cells, macrophages and dendritic cells (DCs) [4], [5]. The CAFs play an important role in tumor progression and this is supported by the fact that many tumors fail to develop unless the stroma is modified [6] and these cellular modifications are induced in a paracrine manner by adjacent tumor cells [7], [8]. Proinflammatory factors such as IL-1, TNF-α, and COX-2 induce the expression of inflammatory genes in CAFs and immune cells present in the tumor [4], [9].

Inflammation is strongly connected to most types of cancer and involve activation of oncogenes and/or inactivation of tumor suppressor genes that influence the proinflammatory transcriptional programs by the malignant cells [10]. In the case for PDAC, several factors have been shown to be involved in tumor and stroma interactions including CXCL8, TGF-β and metalloproteases [11], [12], [13], all observed in our PDAC-CAF cross talk system [9]. The inflammation in PDAC is to high extent driven by IL-1α, expressed and secreted by the tumor cells and affecting the stroma cells, i.e. CAFs, which produce massive amount of inflammatory and immune regulatory factors both in vitro and in vivo [5], [9]. The signaling event induced by IL-1 is well known and starts with IL-1 binding to and signaling through the IL-1 receptor followed by a subsequent activation of the p38 mitogen activated protein kinase (MAPK) [14]. This occur via the small G protein Ras that becomes associated with IRAK, TRAF6, and TAK-1, which facilitate the p38MAPK activation by IL-1 [15]. In contrast, until very recently the signaling events leading up to the expression of IL-1α by the tumor cells had not been elucidated. Ling et al showed for the first time involvement of the K-Ras mutation in codon 12D in induction of IL-1α expression via the transcription factor AP-1 [16]. Moreover, IL-1α activated NF-*K*β and its target genes IL-1α and p62 to initiate IL-1α/p62 feed forward loops, which induced and sustained the NF-*K*β activity [16]. Dysregulation of Ras pathways is common in cancer as this oncogene is the most frequently mutated in human cancers and contribute to cancer cell survival [10]. Activating K-Ras mutations are present in nearly all PDACs (up to 90%) and occur very early and are the most frequent mutations in pancreatic cancer, followed by mutation or silencing of p53, p1, and DPC4/smad4 [17], [18]. For pancreatic cancer, K-Ras mutations are a negative prognostic factor after surgery and adjuvant chemoradiation [19]. The mitogen activated protein kinases (extracellular signal-regulated kinase (ERK), Jun N-terminal kinase (JNK), and p38MAPK) are the best characterized signal pathways in transduction of Ras activity and their oncogenic functions are mostly based on their ability to activate AP-1 [20], [21]. Ras/Raf/MAPK pathway is involved in many cellular processes such as cell cycle regulation, wound healing, cell migration, cell growth, division, and differentiation [10].

So far, there is no selective specific inhibitor of K-Ras available for routine clinical use and downstream targets such as MAPKs are then interesting targets for inhibition of K-Ras signaling. ERK, JNK, and p38MAPK are three major MAPKs and have key roles in inflammation, tissue homeostasis, proliferation, differentiation, migration and survival of cells. ERK is activated by mitogens, whereas JNK and p38MAPKs are activated by cellular stress for instant via the K-Ras signaling pathway. The p38MAPK signaling has been shown to affect proliferation, differentiation, and migration and is associated with cancers both in human and mouse [20].

The aim of this study was to examine the signaling cascade involved in the induction of IL-1α expression in PDAC. We hypothesized that tumor cells creates an inflammatory microenvironment by inducing their expression of IL-1α through down-stream targets of mutated K-Ras and deciphering this could be of relevance to determine targets for treatment.

We found that the p38MAPK, activated by the K-Ras signaling pathway, to be involved in the expression of IL-1α by PDAC cells, as blocking this pathway decreased both the gene and protein expression of IL-1α. Blockage of the p38MAPK signaling in PDAC also dampened the ability of the tumor cell to induce inflammation in CAFs and CAFs ability to enhance tumor cell migration. Noteworthy, the IL1-α autocrine signaling regulated the migratory capacity in PDAC cells. Taken together, the blockage of signaling pathways leading to IL-1α expression and/or neutralization of IL1-α in the PDAC microenvironment should be taken into consideration as possible treatment or complement to existing treatment of this cancer.

## Results

### p38MAPK/ERK is Involved in Tumor Cell Associated IL-1α Expression

**Figure 1:**
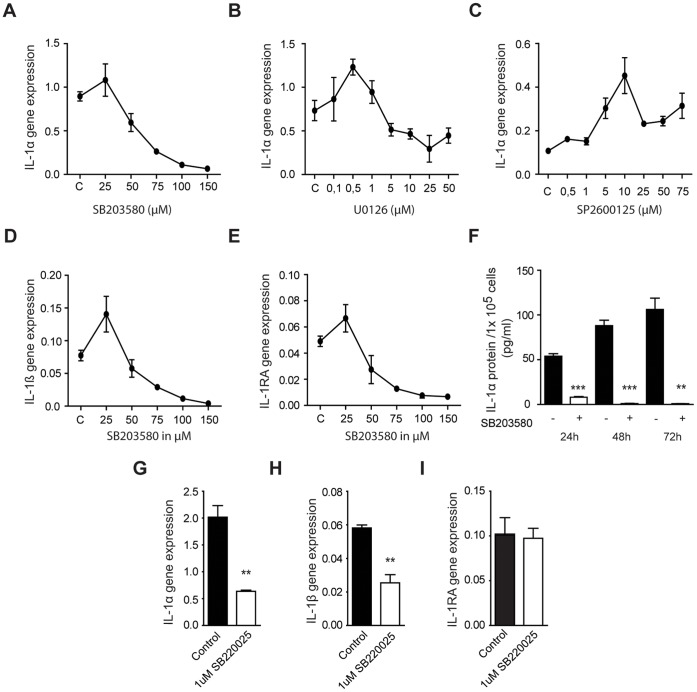
Blockage of p38MAPK signaling decreased the expression of IL-1α in primary PDAC cells. (**A–E**) The primary PDAC cell line PC013 was cultured for 24 h in 1% FBS medium supplemented with vehicle (DMSO) or different concentrations (0–150 µM) of the p38MAPK inhibitor SB203580, the ERK inhibitor U0126 (0–50 µM), or the JNK inhibitor SP600125 (0–75 µM). The mRNA expression levels of (**A–C**) IL-1α, (**D**) IL-1β, and (**E**) IL-1RA were analyzed using qRT-PCR. (**F**) PC013 cells were lysed after 24 h, 48 h, and 72 h after incubation with 100 µM SB203580 or vehicle and IL-1α was measured by ELISA and the result presented in pg/ml per 1×10^5^ cells. (**G–I**) To confirm the effect of SB203580, PC013 cells were treated for 24 h with SB220025, another p38MAPK inhibitor, and the gene expression levels of (**G**) IL-1α, (**H**) IL-1β and (**I**) IL-1RA were analyzed and compared to vehicle. * = P<0.05, ** = P<0.005, and *** = P<0.001.

The IL-1α positive primary PDAC cell line PC013 [9] cultured in 1% FCS were exposed to 0–150 µM p38MAPK (SB203580) inhibitor, 0–50 µM ERK (U0126) inhibitor, and 0–75 µM JNK (SP600125) inhibitor for 24 h. We assessed if the inhibitors asserted any negative effect on cell viability and found no effects on the cell viability. The expression levels of IL-1α mRNA decreased after exposure to the p38MAPK inhibitor, while only a minor decrease was seen after ERK inhibitor, while the JNK inhibitor slightly induce IL-1α expression **(** **Figure 1A–C** **)**. 100 µM of p38MAPK inhibitor SB203580 was found to be optimal to use for subsequent experiments. The mRNA expression levels of IL-1β and IL-1RA decreased after exposure to the p38MAPK inhibitor **(** **Figure 1D–E** **)** and had similar expression curves as IL-1α. We have previously shown that the IL-1α positive PDAC cell lines secrete IL-1α protein and this is in accordance with findings from other tumor cell lines [22]. In addition, our primary PDAC cell lines do not express the IL-1β protein only the mRNA [9] and therefore did we only examining the IL-1α protein expression and found that it was significantly decreased after 24 h (p<0.005), 48 h (p<0.005), and 72 h (p = 0.001) treatment with p38MAPK inhibitor compared to vehicle treated cells **(** **Figure 1F** **)**. We had to use high concentration of SB303580 to block IL-1α and as this inhibitor is known to also work on other MAPKs at high concentration did we confirm the role of p38MAPK in the regulation of tumor cell associated IL-1α by using SB220025, which is considered to be a very specific inhibitor of p38 MAPK than SB203580. This inhibitor significantly reduced the mRNA expression of IL-1α (P = 0.003) and IL-1β (P = 0.003), but did not affect the expression levels of IL-1RA **(** **Figure 1G–I** **)**. In addition, we found that p38 MAPK and ERK was phosphorylated in our primary PDAC cell lines PC013, PC065 and that the inhibition of p38 MAPK with SB220025 did not reduce the phosphorylation of this MAPK, whereas the phosphorylation of ERK was reduced by the ERK inhibitor (data not shown). The phosphorylation of p38 MAPK is in accordance with previous findings for pancreatic cancer cell lines [23], [24], which indicate that this pathway is constitutively activated in PDAC tumor cells.

### The p38MAPK Inhibition had Different Effects on PDACs and CAFs Inflammatory Profiles

**Figure 2:**
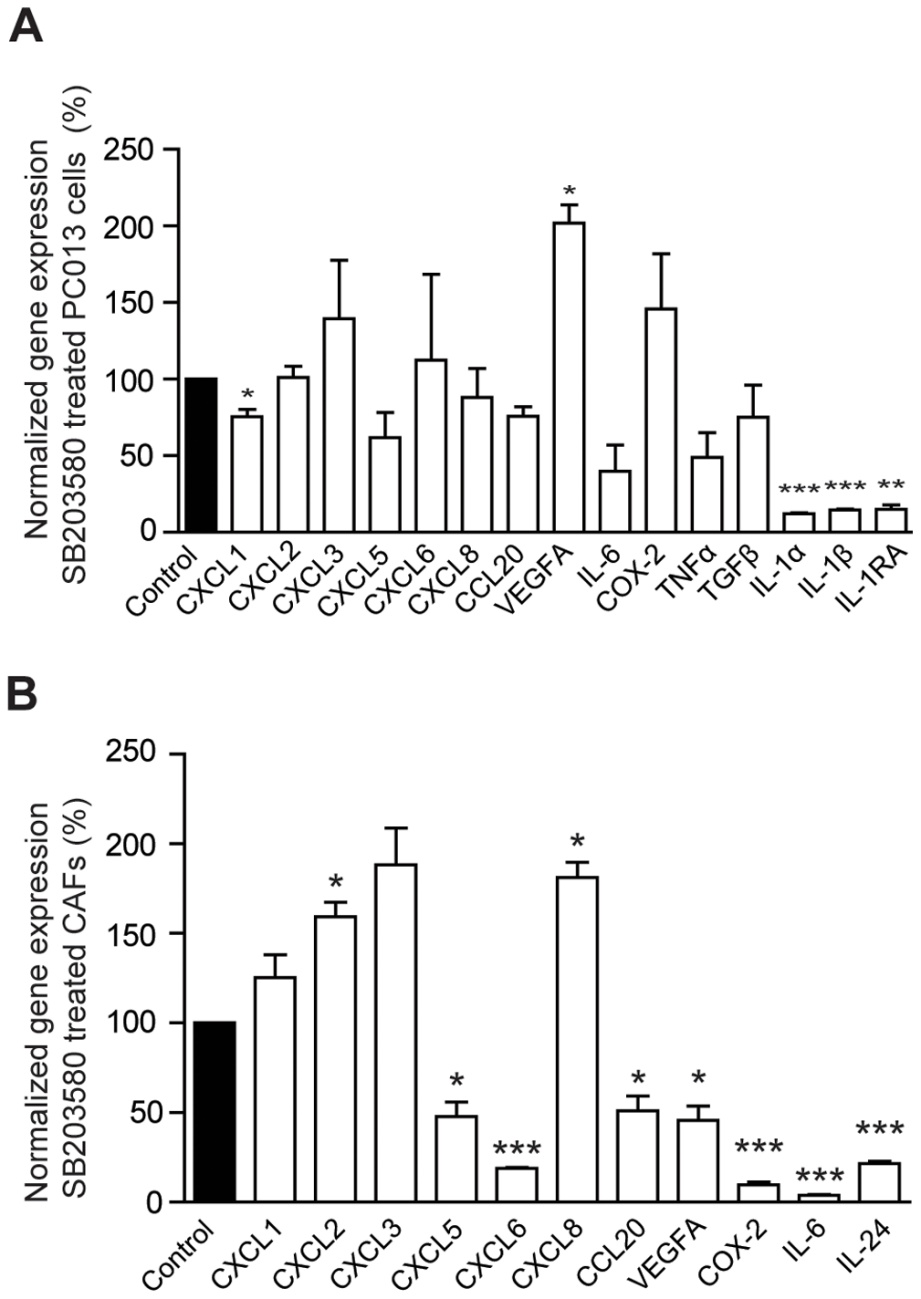
Inhibition of p38MAPK signaling affects the inflammatory profile in PDAC cells and CAFs. The primary PDAC cell line PC013 (**A**) and primary CAFs (**B**) obtained from one PDAC patient were cultured for 24 h with 100 µM of the p38 inhibitor SB203580 in 1% FBS medium. The gene expression levels for CXCL1, CXCL2, CXCL3, CXCL5, CXCL8, CCL20, VEGFA, IL6, COX-2, TNFα, TGFβ, IL24, IL-1α, IL-1β, and IL-1RA were analyzed by qRT-PCR and normalized to vehicle. * = P<0.05, ** = P<0.005, and *** = P<0.001.

The p38MAPK inhibitors had in our study significantly inhibitory effects on PDAC associated IL-1α expression and the p38MAPK pathway have previously been found to be involved in the regulation of chronic inflammation [20]. To examine the regulatory role of p38MAPK on other inflammatory factors we investigated the direct effects of SB203580 on PC013 cells and CAFs. The results for the primary PDAC cell line PC013 showed significant reduced levels of IL-1α (P<0.001), IL-1β (P<0.001), IL-1RA (P = 0.001), and CXCL1 (P = 0.04), whereas CCL20, and TNF-α levels were decreased but not significant **(** **Figure 2A** **)**. Levels of VEGFA, CXCL3 and COX-2 increased but only VEGFA to a significant level (P = 0.01) **(** **Figure 2A** **)**. Blockage of p38MAPK pathway in CAFs for 24 h resulted in increased mRNA expression levels of CXCL2 (P = 0.02), CXCL8 (P = 0.01) and CXCL3 (P = 0.05) in CAFs **(** **Figure 2B** **)**. Furthermore, the CAF gene expression levels of CXCL5 (P = 0.024), CXCL6 (P<0.001), CCL20 (P = 0.03), VEGFA (P = 0.02), COX-2 (P<0.001), IL-6 (P<0.001), and IL-24 (P<0.001) decreased after blockage of p38MAPK **(** **Figure 2B** **)**. This clearly shows that the p38MAPK signaling regulates the expression of inflammatory factors differently in PDAC cells compared to CAFs.

### Tumor Cell p38MAPK Signaling and IL-1 Regulation Affects the Tumor-CAF Crosstalk

**Figure 3:**
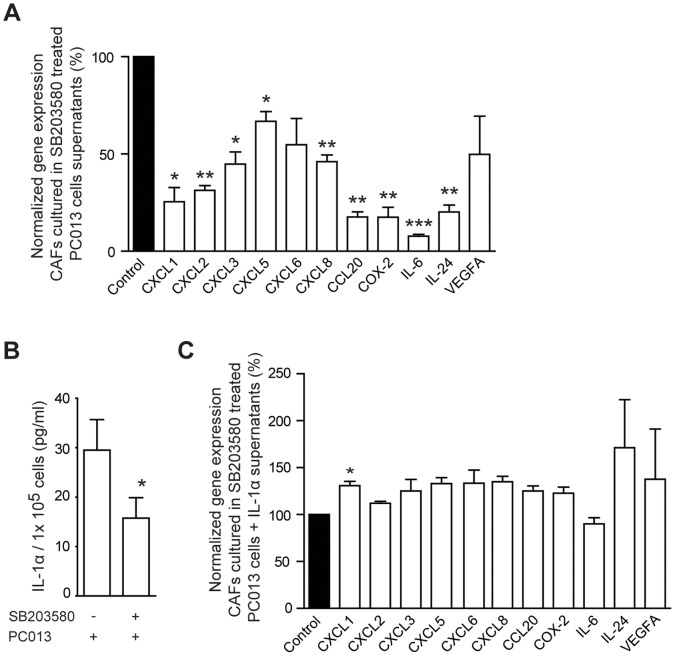
Down regulation of p38MAPK signaling decreased the levels of tumor cell associated IL-1α and the inflammatory profile in CAFs. The primary PDAC cell line PC013 was cultured in 1%FBS medium containing 100 µM SB203580 or vehicle for 72 h. The 1%FBS medium was replenished and the cells cultured for another 48 h before the supernatant was transferred to CAFs. The CAFs were cultured for 72 h. The inflammatory gene profile for the CAFs (A) was analyzed by normalizing the effects of SB203580 to vehicle. The supernatant are referred to as (PC013/vehicle/CAF) and (PC013/SB203580/CAF). IL-1α was measured in the supernatants from SB203580 and vehicle treated PC013 cells by ELISA and the result is presented in pg/ml per 1×10^5^ cells (B). The IL-1α levels in the supernatants from the SB203580 treated PC013 cells were equalized to the control supernatants by adding rhIL-1α. The IL-1α normalized supernatants were added to CAFs and incubated for 72 h. The inflammatory gene profile was analyzed by normalizing the IL-1α supernatants to vehicle supernatants (C).

IL-1α has previously been established as an important factor involved in the communication between tumor cells and CAFs in PDAC [9]. Our findings of an inhibiting role of p38MAPK inhibitors on PDAC tumor cell IL-1α expression could have the potential to affect the crosstalk between tumor cells and CAFs. We examined the inflammatory profile of CAFs conditioned with supernatants derived from PC013 first pretreated with p38MAPK inhibitor for 72 h than recultured 48 h without inhibitor. The conditioned CAFs showed significantly decreased gene levels of CXCL1 (P = 0.01), CXCL2 (P = 0.001), CXCL3 (P = 0.012), CXCL5 (P = 0.023), CXCL8 (P = 0.004), CCL20 (P = 0.001), COX-2 (P = 0.004), IL-6 (P<0.001), and IL-24 (P = 0.002) **(** **Figure 3A** **)** compared to supernatants from vehicle treated PC013 cells. To relate these findings in CAFs to tumor cell associated IL-1α, as a downstream result of p38MAPK signaling, we analyzed the IL-1α levels in the PC013 supernatants 2 days after the exposure to p38MAPK inhibitor and found significantly reduced levels of IL-1α (P = 0.038) **(** **Figure 3B** **)**. Next was the concentration of IL-1α in the p38MAPK pretreated PC013 derived supernatants returned to original levels by addition of exogenous rhIL-1α and used to treat CAFs for 48 h. The CAF gene profile showed increased levels of inflammatory factors **(** **Figure 3C** **)**. These results indicate an important role for p38MAPK in regulating PDAC cell-CAF crosstalk through the induction/upregulation of IL-1α expression in PDAC.

### The Migratory Properties of the Tumor Cells is Enhanced by the Autocrine and Paracrine Effects Exerted by IL-1α on PDAC and CAFs

**Figure 4:**
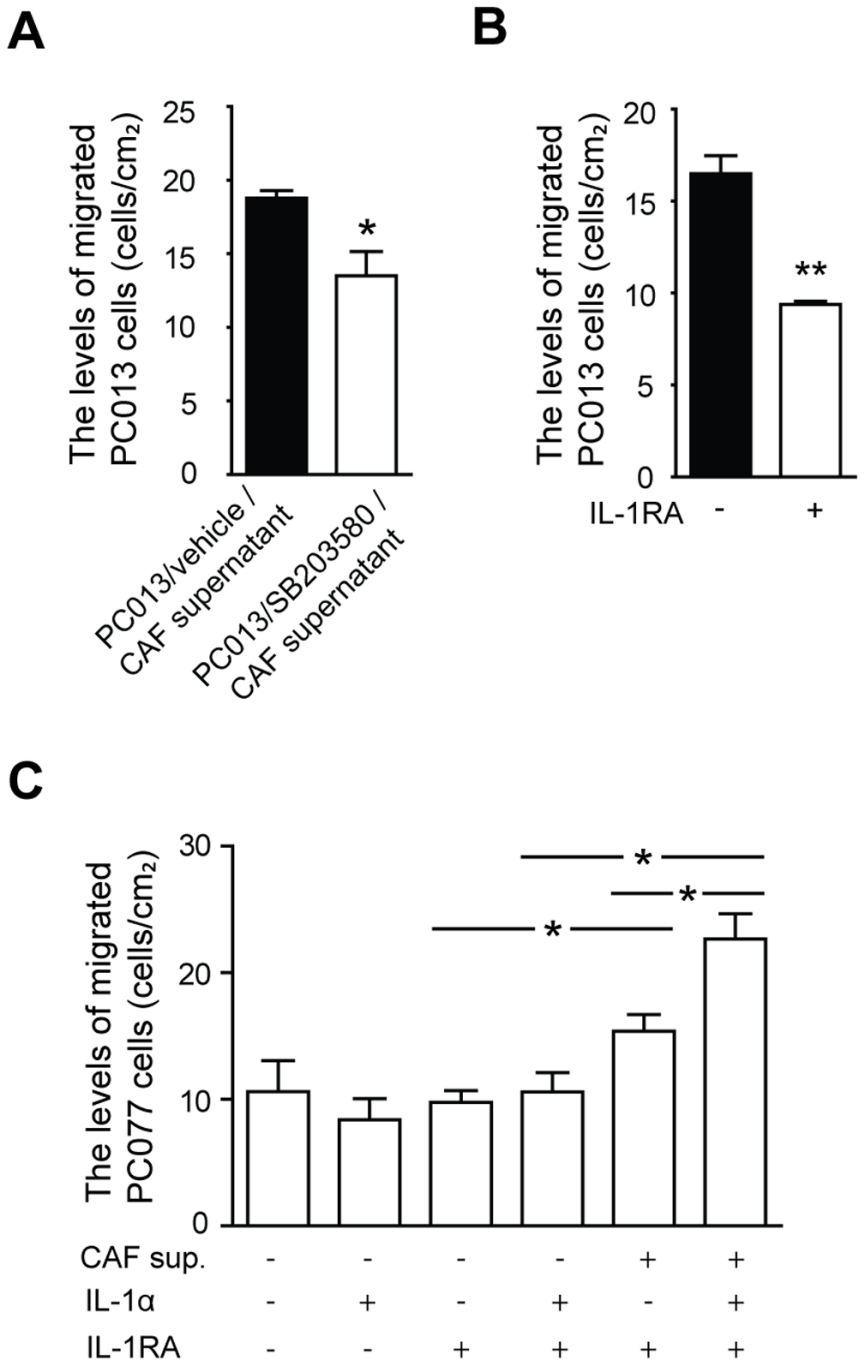
The migratory properties of the tumor cells are enhanced by the autocrine and paracrine effects exerted by IL-1α on PDAC and CAFs. IL-1α positive PC013 and IL-1α negative PC077 cells were starved in 1%FBS overnight and seeded on Transwell filters pre coated with 10 µg/ml fibronectin. PC013 cells were incubated for 40 h in PC013/vehicle/CAF and PC013/SB203580/CAF supernatants (A), and 100 ng/ml IL-1RA (B). IL-1α negative PC077 cells were cultured for 72 h in 1% FBS medium containing mock, 500 pg/ml IL-1α, 100 ng/ml IL-1RA alone, 100 ng/ml IL-1RA together with either 500 pg/ml IL-1α, CAF supernatant (7 days), or IL-1α (500 pg/ml) activated CAF supernatant (7 days) (C). The adherent cells were fixed in 4% formaldehyde, visualized using crystal violet and quantified manually using 10× magnification in an inverted microscope. The result is presented as cells/cm_2_. * = P<0.05, ** = P<0.005, and *** = P<0.001.

We further investigated if the decreased level of inflammatory factors in CAFs, treated with supernatants derived from p38MAPK signaling inhibited PC013 cells, affected the ability of the tumor cells to migrate. PC013 cells exposed to supernatants derived from CAFs cocultured with PC013 cells pretreated with p38MAPK (IL-1α low) had significantly decreased migration compared to cells exposed to supernatants derived from CAFs cocultured with untreated PC013 cells (P = 0.038) **(** **Figure 4A** **)**. The decreased levels of IL-1α in PC013 cells exposed to p38MAPK inhibitor should reduce IL-1α autocrine feedback and modulate the functions such as tumor cell mobility. We investigated this by neutralized IL-1α with rhIL-1RA and found a significantly reduced migration of PC013 cells (P = 0.002) **(** **Figure 4B** **)**. To in depth elucidate the effect of IL-1α on tumor cell migration we used PC077, a primary PDAC cell line that is IL-1α negative [9]. In addition, rhIL-1RA was added to all groups in this experiment to eliminate any direct effects of IL-1α on the tumor cell migration. Supernatants from untreated CAFs (P = 0.025) significantly enhanced the migration of IL-1RA treated PC077 cells, while supernatants from exogenous IL-1α activated CAFs even further increased migration of PC077 cells compared to both IL-1RA/IL-1α (P = 0.009) treated, IL-1α treated, and untreated CAF supernatants (P = 0.038) **(** **Figure 4C** **)**. Moreover, no difference was found between rhIL-1RA and IL-1α/rhIL-1RA treated PC077 cells. Taken together, this indicates that IL-1α not only induce autocrine direct effects on cancer cell migration, but also enhance migration through paracrine signaling and activation of CAFs that obtain the ability to stimulate tumor cell migration by altering the tumor cell phenotype, including increased expression of metalloproteases and factors involved in epithelial mesenchymal transition (work in progress).

### rhIL-1RA in Combination with p38MAPK Inhibitor Effectively Reduced the Levels of Inflammatory Factors Induced in CAFs by PDAC Cells

**Figure 5:**
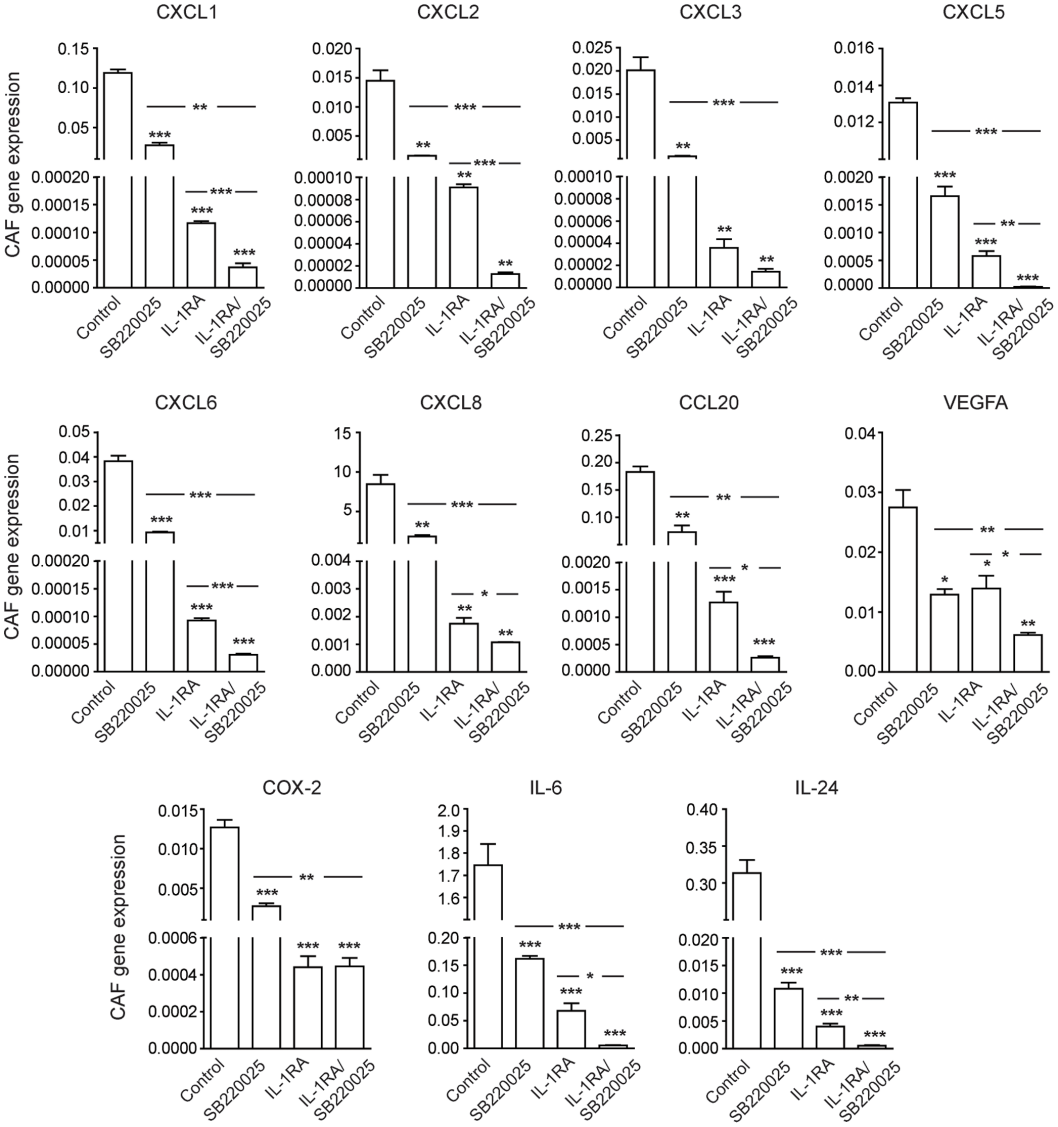
Combination therapy with a p38MAPK inhibitor and IL-1RA was superior to single therapy in inhibiting the PDAC-CAF crosstalk induced inflammation. The PDAC cell line PC013 was cocultured with primary CAFs using cell culture inserts for 5 days in 1% FBS medium supplemented with p38MAPK inhibitor SB220025 (1 µM) or rhIL-1RA (10 µg/ml) or a combination of these agents. CAFs inflammatory gene expression profile after SB220025 and rhIL-1RA single or combination treatment were compared to cocultured vehicle treated CAFs The combination of the two agents were also compared to single agent treatment and the result is shown as relative gene expression. * = P<0.05, ** = P<0.005, and *** = P<0.001.

IL-1RA has previously been shown to decrease the levels of inflammatory factors in both single and CAFs cocultured with PDAC cells [9]. Here we examined the effects treatment with IL-1RA and p38MAPK inhibitor alone or as a combination therapy had on PC013 and CAF cocultures. Blockage of p38MAPK signaling significantly decreased the levels of CXCL1, CXCL5, CXCL6, COX-2, IL-6, and IL-24 (P<0.001), CXCL2, and CCL20 (P = 0.002), CXCL3 (P = 0.003), CXCL8 (P = 0.004), and VEGFA (P = 0.009), **(** **Figure 5** **)** in CAFs cocultured with PDAC cells compared to untreated controls (2–29 fold decease) **(** **Table 1** **)**. Neutralization of IL-1 by rhIL-1RA as a single agent drastically reduced the levels of all the inflammatory factors (2–4876 fold) compared to untreated cocultured CAFs (CXCL1. CXCL5, CXCL6, CCL20, IL-6, IL-24 and COX-2 (P<0.001), CXCL2, CXCL3, and CXCL8 (P = 0.002), and VEGFA (P = 0.02)) **(** **Figure 5** **and** **Table 1** **)**. The combination of p38MAPK inhibitor and rhIL-1RA decreased the inflammation 4.5–7895 folds compared to untreated controls (CXCL1, CXCL2, CXCL5, CXCL6, CCL20, COX-2, IL-6, and IL-24 (P<0.001), CXCL3, CXCL8, and VEGFA (P = 0.002)) **(** **Figure 5** **and** **Table 1** **)**. Moreover, the combination therapy decreased the levels of the inflammatory factors compared to rhIL-1RA treated cocultured CAFs (CXCL1, CXCL2, CXCL6 (P<0.001), CXCL3 (P = 0.06), CXCL5 (P = 0.003), CXCL8 (P = 0.04), CCL20 (P = 0.007), VEGFA (P = 0.02), IL-6 (P = 0.009) and IL-24 (P = 0.003)) **(** **Figure 5** **)**.

**Table 1:** CAF inflammatory profile after single and combination therapy with rhIL-1RA and p38MAPK inhibitor SB220025.

	CXCL1	CXCL2	CXCL3	CXCL5	CXCL6	CXCL8	CCL20	VEGFA	COX-2	IL-6	IL-24
**SB220025**	4	9	14	8	4	5	2	2	5	11	29
**rhIL-1RA**	1016	159	564	22	412	4876	144	2	29	26	79
**SB220025/rhIL-1RA**	3214	1131	1430	684	1236	7892	695	5	28	325	612

The data is presented as fold decrease compared to untreated control CAF.

## Discussion

We have demonstrated that the p38MAPK signaling pathway is involved in the expression of IL-1α by PDAC cells and that IL-1α initiates a change in the PDAC phenotype and properties, making the tumor cells more prone to migrate. The p38MAPK signaling in tumor cells were shown to be involved in the upregulation of inflammation in CAFs via the induction of IL-1α and to our knowledge has this not been shown previously. Our previous finding that IL-1α overexpression correlated with poor survival in PDAC patients was confirmed in the study by Ling et al [16]. These findings suggest that inhibition of IL-1α expression and activity in PDAC will drastically decrease the levels of inflammatory factors in CAFs, and reducing and/or neutralizing the effects of IL-1α could have the potential to reduce tumor spread and improve the clinical outcome for the patients [5], [9].

The signaling pathways/events giving rise to constitutive expression of IL-1α have been elusive. A recent study by Ling et al [16] using a mouse model where mutation in K-Ras G12D was used to induce PDAC showed that this activating K-Ras mutation induced activation of NF-kB, which was required for PDAC development, and expression of IL-1α in the tumor cells and that this create a intrinsic inflammatory response that promote a pro-tumorigenic microenvironment through the expression of inflammatory mediators, e.g. cytokines such as IL-1α [16]. The proposed mechanism for NF-kB activation by K-Ras is through AP-1 induced IL-1α overexpression [16]. The expression of IL-1α by tumor cells is detected in 90% of the PDAC patients [9] and could correlate to mutations in the oncogene K-Ras as they also are present in up to 90% of the PDAC cases [25], [26], [27]. These findings clearly demonstrate the role of inflammation in the development of PDAC. As all attempts of developing a drug that directly blocks the mutated K-Ras oncogene has been unsuccessful [28], did we target downstream factors activated by K-Ras. The signaling cascades through K-Ras lead to many different events inside the cell and several pathways, i.e. RAS/RAF/MAPK RAS/P13K/AKT, are well characterized [29]. In response to cellular stress and cytokine stimulation mediated through K-Ras do p38MAPK kinases (MMK3 and MKK6) and JNK kinases (MKK4 and MKK7) phosphorylate p38MAPK and JNK, respectively [15].

PDAC have a very elevated expression of multiple inflammatory genes and the main cell in the tumor stroma, i.e. CAFs, are the major producers of these factors [5], [9]. According to our own data and the study by Ling et al [16], IL-1α is the pro-inflammatory factor responsible for inducing the expression of the inflammation. We found that the IL-1α gene and protein expression by primary PDAC cell lines was down regulated only when the signaling through p38MAPK was blocked. This clearly show that signaling trough activated p38MAPK highly influence the IL-1α expression and also eliminates ERK and JNK as contributors to the regulation of IL-1α in PDAC cells. In addition, the inhibition of p38MAPK in PDAC lead to a modulated gene profile for several inflammatory genes, some factors decreased whereas others increased.

Interfering with the p38MAPK signaling pathway also directly affected the inflammatory profile of primary CAFs, but the profile differed from the one induced in PDAC, explained by other pathways affected in CAFs compared to PDAC cells. The CAFs play an important role in tumor progression and this is supported by that many tumors fail to develop unless the stroma is modified [6] and these changes are induced in a paracrine manner by adjacent tumor cells [7], [8]. The paracrine action of IL-1α, expressed by the tumor cells, seems to be important in the case for developing PDAC [16]. Both soluble and cellular factors are involved in tumor and stroma interactions, including CXCL8, TGFβ, and metalloproteases [9], [11], [12], [13]. The crosstalk between PDAC cells and CAFs induce a high level of inflammation and it was significantly diminished when the PDAC had been pretreated with p38MAPK inhibitors. The down regulation of the inflammatory profile in CAFs was due to the reduced levels of IL-1α expressed by the PDAC cells as replenishing this proinflammatory factor restored the inflammatory responses.

The expression of IL-1α by PDAC affects the tumor cell function, making them more prone to migrate as neutralizing of IL-1α with IL-1RA decreased the migratory capacity. In addition to the autocrine effects of IL-1α on tumor cell migration, the protein also acts in a paracrine manner by promoting a CAF phenotype supporting the tumor cell migration. Induction of IL-1α expression in PDAC cell lines enhance their ability to form metastasis and invade tissue in orthotopic mouse models and in vitro experimental systems [2], [30]. Similar effects with increased ability to metastasize has also been described for tumor cells expressing IL-1β [31], [32].

The PDAC creates a microenvironment that makes this tumor hard to treat and reducing the inflammation could be one way to improve this. Down regulation of IL-1α expression by p38MAPK in combination with IL-1RA more or less abolished the inflammation in PDAC and CAFs cocultures. Consequently, the inhibition of signaling pathways leading to IL-1α expression and/or neutralization of IL-1α in the PDAC microenvironment should be taken into consideration as a possible treatment or as a complement to existing treatments of this cancer.

## Materials and Methods

### Ethical Statement

The primary PDAC cell lines PC013 and PC077, and primary CAFs were propagated from PDAC tumor tissue biopsies as described elsewhere [9]. The study protocol and patient consent documents were approved by the Regional Ethics Committee in Linköping, Sweden (Dnr. M38-06). The consent was written and obtained from all participants involved in the study.

### PDAC and CAF Cell Lines

The primary PDAC cell lines PC013 and PC077, and primary CAFs were cultured in RPMI 1640 (Fisher Scientific, Pittsburgh, PA), supplemented with 1% FCS (Invitrogen), 2 mM HEPES (Invitrogen), 30 µg/ml Gentamycin (Invitrogen) (R10). For all assays, at 6×10^5^ PC013 and PC077 cells were seeded per well and cultured for 24 h before adding the different inhibitors.

### Culturing PDAC Cells and CAFs with p38MAPK, ERK and JNK Inhibitors

PC013 cells were cultured in R10 containing vehicle (DMSO), 0.1–150 µM p38MAPK inhibitor SB203580 (Cayman Chemicals, US), p38MAPK inhibitor SB220025, ERK inhibitor UO126, or JNK inhibitor SP2600125 (SigmaAldrich, Sweden) for 24–72 h. In other sets of experiments, PC013 cells were cultured with 100 µM p38MAPK inhibitor SB203580 or vehicle (DMSO) for 72 h, washed 3 times and recultured in 2 ml R10 for 48 h. The 48 h supernatants were transferred and used to culture CAFs for 72 h. The PC013-SB203580-CAF (C20) and PC013-vehicle-CAF (C30) conditioned medium were normalized to number of cells/group (1×10^5^ cells/ml medium) to adjust for differences in the total amount of cells. To confirm the role of IL-1α in tumor cell-CAF cross-talk, the levels of IL-1α was measured in the supernatants from SB203580 and vehicle treated PC013 cells and equalized with rhIL-1α (R&D Systems, UK). The IL-1α normalized medium was added to CAFs for 72 h. The cells were lysed with RLT buffer (Qiagen) and RNA prepared as described elsewhere [9].

### Treatment of PDAC and CAFs with p38MAPK Inhibitor and IL-1RA

The effects of p38 inhibitor (SB220025) and IL-1RA on tumor cell/CAF cross-talk were investigated and PC013 cells and CAFs were cocultured in inserts (0.4 µm) (BD, USA) for 5 days in R10 medium supplemented with rhIL-1RA (10 µg/ml) (Kineret 100 mg, Biovitrum AB) and/or 1 µM SB220025.

### RNA Extraction and Quantification

Total RNA purification and, cDNA was prepared as previously described [9]. Quantitative PCR was performed with Fast SYBR Green Master Mix (Version 09/2007; Applied Biosystems, Foster City, CA) on 7900 Fast Real-Time PCR system with 7900 System SDS 2.3 Software (Applied Biosystems). In the negative controls cDNA were replaced by distilled water. Specific primers for CXCL8, CCL20, IL-1α, IL-1β, IL-6, IL-24, IL-1RA, TGFβ, VEGF-A, (CyberGene AB), and COX-2 (Invitrogen) were used. Glyceraldehyde-3-phosphate dehydrogenase (GAPDH) (CyberGene AB) and actin were utilized as housekeeping genes. The primers were designed using Primer Express (Applied Biosystems) if not otherwise indicated. Real-time PCRs for the detection of CXCL chemokines and TNFα were performed using TaqMan® Gene Expression Assays (Applied BioSystems) according to the manufacturer’s protocol. All reactions were performed in triplicates including none-template controls and endogenous control probes. FAM conjugated, gene specific assays were Hs00236937_m1 (CXCL1), Hs00236966_m1 (CXCL2) Hs00171061_m1 (CXCL3), Hs00171085_m1 (CXCL5), Hs00237017_m1 (CXCL6), and Hs99999043_m1 (TNFα). The results were analyzed using the ΔΔCt method [33] and presented as either normalized data or as relative gene expression.

### ELISA

Supernatants were collected and cells harvested and counted before lysis with lysis buffer pH 7.5. The lysates and supernatants were analyzed for the concentration of IL-1α by ELISA (Nordic Biosite, Sweden) according to the manufacturers’ protocols. This ELISA measures precursor, secreted, and membrane-associated forms of IL-1α. The levels of IL-1α are presented as pg/1×10^5^ cells.

### Migration Assay

Transwell migration assay were conducted as described elsewhere [34]. Briefly, PC077 and PC013 were starved in 1% FBS overnight and seeded on 6-well plates Transwell filters (Costar) (8 µm pore size) precoated with 10 µg/ml fibronectin (Sigma, St. Louis, MO). PC077 cells were incubated for 40 h in 1% FBS medium containing 100 ng/ml IL-1RA and either 500 pg/ml IL-1α, CAF supernatant (7 days), or IL-1α (500 pg/ml) activated CAF supernatant (7 days). PC013 cells were incubated for 40 h in 1% FBS and/or 100 ng/ml IL-1RA, C20, and C30 conditioned medium. The upper compartment was removed and the medium discarded. The adherent cells were washed in PBS, fixed in 4% formaldehyde and visualized using crystal violet (SigmaAldrich, Sweden). The sample identification was blinded and all attached cells were manually counted using 10× magnifications in an inverted microscope (Leica). The data was obtained from 3 experiments (mean ± standard error) and presented as the mean cells/cm^2^.

### Statistical Analysis

The statistical analysis was performed with GraphPad Prism 5 (GraphPad Software), P<0.05 was considered statistically significant and error bars throughout indicate standard error of the mean (SEM). The data were analyzed using the paired t-.test and unpaired t-test was used for normalized data.

## References

[1] Li D, Xie K, Wolff R, Abbruzzese JL (2004) Pancreatic cancer. Lancet 363: 1049–1057. 15051286 10.1016/S0140-6736(04)15841-8

[2] Melisi D, Niu J, Chang Z, Xia Q, Peng B, et al (2009) Secreted interleukin-1alpha induces a metastatic phenotype in pancreatic cancer by sustaining a constitutive activation of nuclear factor-kappaB. Mol Cancer Res 7: 624–633. 19435817 10.1158/1541-7786.MCR-08-0201 PMC2856954

[3] Korc M (2007) Pancreatic cancer-associated stroma production. Am J Surg 194: S84–86. 17903452 10.1016/j.amjsurg.2007.05.004 PMC2094116

[4] Esposito I, Menicagli M, Funel N, Bergmann F, Boggi U, et al (2004) Inflammatory cells contribute to the generation of an angiogenic phenotype in pancreatic ductal adenocarcinoma. J Clin Pathol 57: 630–636. 15166270 10.1136/jcp.2003.014498 PMC1770337

[5] Tjomsland V, Niklasson L, Sandstrom P, Borch K, Druid H, et al (2011) The desmoplastic stroma plays an essential role in the accumulation and modulation of infiltrated immune cells in pancreatic adenocarcinoma. Clin Dev Immunol 2011: 212810. 22190968 10.1155/2011/212810 PMC3235447

[6] Franco OE, Shaw AK, Strand DW, Hayward SW (2011) Cancer associated fibroblasts in cancer pathogenesis. Semin Cell Dev Biol 21: 33–39. 10.1016/j.semcdb.2009.10.010 PMC2823834 19896548

[7] Sato N, Maehara N, Goggins M (2004) Gene expression profiling of tumor-stromal interactions between pancreatic cancer cells and stromal fibroblasts. Cancer Res 64: 6950–6956. 15466186 10.1158/0008-5472.CAN-04-0677

[8] Somasundaram R, Herlyn D (2009) Chemokines and the microenvironment in neuroectodermal tumor-host interaction. Semin Cancer Biol 19: 92–96. 19049876 10.1016/j.semcancer.2008.11.002 PMC2806055

[9] Tjomsland V, Spangeus A, Valila J, Sandstrom P, Borch K, et al (2011) Interleukin 1alpha sustains the expression of inflammatory factors in human pancreatic cancer microenvironment by targeting cancer-associated fibroblasts. Neoplasia 13: 664–675. 21847358 10.1593/neo.11332 PMC3156657

[10] Pylayeva-Gupta Y, Grabocka E, Bar-Sagi D (2011) RAS oncogenes: weaving a tumorigenic web. Nat Rev Cancer 11: 761–774. 21993244 10.1038/nrc3106 PMC3632399

[11] Sato N, Fukushima N, Maehara N, Matsubayashi H, Koopmann J, et al (2003) SPARC/osteonectin is a frequent target for aberrant methylation in pancreatic adenocarcinoma and a mediator of tumor-stromal interactions. Oncogene 22: 5021–5030. 12902985 10.1038/sj.onc.1206807

[12] Saad S, Gottlieb DJ, Bradstock KF, Overall CM, Bendall LJ (2002) Cancer cell-associated fibronectin induces release of matrix metalloproteinase-2 from normal fibroblasts. Cancer Res 62: 283–289. 11782389

[13] Dong Z, Nemeth JA, Cher ML, Palmer KC, Bright RC, et al (2001) Differential regulation of matrix metalloproteinase-9, tissue inhibitor of metalloproteinase-1 (TIMP-1) and TIMP-2 expression in co-cultures of prostate cancer and stromal cells. Int J Cancer 93: 507–515. 11477554 10.1002/ijc.1358

[14] Freshney NW, Rawlinson L, Guesdon F, Jones E, Cowley S, et al (1994) Interleukin-1 activates a novel protein kinase cascade that results in the phosphorylation of Hsp27. Cell 78: 1039–1049. 7923354 10.1016/0092-8674(94)90278-x

[15] McDermott EP, O’Neill LA (2002) Ras participates in the activation of p38 MAPK by interleukin-1 by associating with IRAK, IRAK2, TRAF6, and TAK-1. J Biol Chem 277: 7808–7815. 11744690 10.1074/jbc.M108133200

[16] Ling J, Kang Y, Zhao R, Xia Q, Lee DF, et al (2012) KrasG12D-induced IKK2/beta/NF-kappaB activation by IL-1alpha and p62 feedforward loops is required for development of pancreatic ductal adenocarcinoma. Cancer Cell 21: 105–120. 22264792 10.1016/j.ccr.2011.12.006 PMC3360958

[17] Slebos RJ, Hoppin JA, Tolbert PE, Holly EA, Brock JW, et al (2000) K-ras and p53 in pancreatic cancer: association with medical history, histopathology, and environmental exposures in a population-based study. Cancer Epidemiol Biomarkers Prev 9: 1223–1232. 11097231

[18] Hruban RH, Goggins M, Parsons J, Kern SE (2000) Progression model for pancreatic cancer. Clin Cancer Res 6: 2969–2972. 10955772

[19] Brunner TB, Cengel KA, Hahn SM, Wu J, Fraker DL, et al (2005) Pancreatic cancer cell radiation survival and prenyltransferase inhibition: the role of K-Ras. Cancer Res 65: 8433–8441. 16166322 10.1158/0008-5472.CAN-05-0158

[20] Wagner EF, Nebreda AR (2009) Signal integration by JNK and p38 MAPK pathways in cancer development. Nat Rev Cancer 9: 537–549. 19629069 10.1038/nrc2694

[21] Chang L, Karin M (2001) Mammalian MAP kinase signalling cascades. Nature 410: 37–40. 11242034 10.1038/35065000

[22] Ebrahimi B, Tucker SL, Li D, Abbruzzese JL, Kurzrock R (2004) Cytokines in pancreatic carcinoma: correlation with phenotypic characteristics and prognosis. Cancer 101: 2727–2736. 15526319 10.1002/cncr.20672

[23] Takekawa M, Tatebayashi K, Itoh F, Adachi M, Imai K, et al (2002) Smad-dependent GADD45beta expression mediates delayed activation of p38 MAP kinase by TGF-beta. Embo J 21: 6473–6482. 12456654 10.1093/emboj/cdf643 PMC136947

[24] Masui T, Doi R, Mori T, Toyoda E, Koizumi M, et al (2004) Metastin and its variant forms suppress migration of pancreatic cancer cells. Biochem Biophys Res Commun 315: 85–92. 15013429 10.1016/j.bbrc.2004.01.021

[25] Mu DQ, Peng YS, Xu QJ (2004) Values of mutations of K-ras oncogene at codon 12 in detection of pancreatic cancer: 15-year experience. World J Gastroenterol 10: 471–475. 14966900 10.3748/wjg.v10.i4.471 PMC4716963

[26] Tabata T, Fujimori T, Maeda S, Yamamoto M, Saitoh Y (1993) The role of Ras mutation in pancreatic cancer, precancerous lesions, and chronic pancreatitis. Int J Pancreatol 14: 237–244. 8113625 10.1007/BF02784932

[27] Kim ST, Lim do H, Jang KT, Lim T, Lee J, et al (2011) Impact of KRAS mutations on clinical outcomes in pancreatic cancer patients treated with first-line gemcitabine-based chemotherapy. Mol Cancer Ther 10: 1993–1999. 21862683 10.1158/1535-7163.MCT-11-0269

[28] Gysin S, Salt M, Young A, McCormick F (2011) Therapeutic strategies for targeting ras proteins. Genes Cancer 2: 359–372. 21779505 10.1177/1947601911412376 PMC3128641

[29] Meier F, Schittek B, Busch S, Garbe C, Smalley K, et al (2005) The RAS/RAF/MEK/ERK and PI3K/AKT signaling pathways present molecular targets for the effective treatment of advanced melanoma. Front Biosci 10: 2986–3001. 15970553 10.2741/1755

[30] Matsuo Y, Sawai H, Ochi N, Yasuda A, Takahashi H, et al (2009) Interleukin-1alpha secreted by pancreatic cancer cells promotes angiogenesis and its therapeutic implications. J Surg Res 153: 274–281. 18952231 10.1016/j.jss.2008.04.040

[31] Chirivi RG, Garofalo A, Padura IM, Mantovani A, Giavazzi R (1993) Interleukin 1 receptor antagonist inhibits the augmentation of metastasis induced by interleukin 1 or lipopolysaccharide in a human melanoma/nude mouse system. Cancer Res 53: 5051–5054. 8267795

[32] Pantschenko AG, Pushkar I, Anderson KH, Wang Y, Miller LJ, et al (2003) The interleukin-1 family of cytokines and receptors in human breast cancer: implications for tumor progression. Int J Oncol 23: 269–284. 12851675

[33] Livak KJ, Schmittgen TD (2001) Analysis of relative gene expression data using real-time quantitative PCR and the 2(-Delta Delta C(T)) Method. Methods 25: 402–408. 11846609 10.1006/meth.2001.1262

[34] Manes T, Zheng DQ, Tognin S, Woodard AS, Marchisio PC, et al (2003) Alpha(v)beta3 integrin expression up-regulates cdc2, which modulates cell migration. J Cell Biol 161: 817–826. 12771130 10.1083/jcb.200212172 PMC2199360

